# Research progress of GLP-1RAs in the treatment of type 2 diabetes mellitus

**DOI:** 10.3389/fphar.2024.1483792

**Published:** 2025-01-20

**Authors:** Xu He, Wei Zhao, PeiHang Li, YinJiang Zhang, GuoHua Li, HongYu Su, BiNan Lu, ZongRan Pang

**Affiliations:** ^1^ School of Pharmacy, Minzu University of China, Beijing, China; ^2^ Key Laboratory of Ethnomedicine (Minzu University of China), Ministry of Education, Beijing, China; ^3^ Pharmacy Department, People’s Hospital of Dali Bai Autonomous Prefecture, Dali, China; ^4^ Graduate School, Chengde Medical College, Chengde, China

**Keywords:** GLP-1, GLP-1RAs, T2DM, glucose, mechanism

## Abstract

Glucagon-like peptide-1 (GLP-1) is a 30-amino acid intestinal insulin-stimulating factor, which is mainly secreted by L cells in the distal ileum and colon. It has various physiological functions, such as promoting insulin secretion and synthesis, stimulating β-cell proliferation, inducing islet regeneration, inhibiting β-cell apoptosis and glucagon release, delaying gastric emptying and controlling appetite, etc. It plays a role through a specific GLP-1 receptor (GLP-1R) distributed in many organs or tissues and participates in the regulation of glucose homeostasis in the body. GLP-1 receptor agonists (GLP-1RAs) has the similar physiological function of GLP-1. Because of its structural difference from natural GLP-1, it is not easy to be degraded by dipeptidyl peptidase-4 (DPP-4), thus prolonging the action time. GLP-1RAs have been recognized as a new type of hypoglycemic drugs and widely used in the treatment of type 2 diabetes mellitus (T2DM). Compared with other non-insulin hypoglycemic drugs, it can not only effectively reduce blood glucose and glycosylated hemoglobin (HbA1c), but also protect cardiovascular system, nervous system and kidney function without causing hypoglycemia and weight gain. Therefore, GLP-1RAs has good application prospects and potential for further development.

## 1 Introduction

The idea that substances produced by duodenal mucosa could stimulate and initiate pancreatic endocrine, thereby reducing the amount of urinary glucose in patients with diabetes, was first proposed in 1906 (Moore, 1906). Elrick et al. have shown that compared with intravenous glucose, the increase of plasma insulin level after oral glucose was greater, and the high level of insulin was maintained for a longer time, which provided a basis for intestinal factors to stimulate insulin secretion (Elrick et al., 1964). The enterogenic factor which has the function of reducing blood glucose is named “enteropagin” (Edgard and Jean, 2017). The first identified enteropagin is glucose-dependent insulin-stimulating peptide (GIP). However, immune neutralization of GIP or removal of GIP from intestinal extracts does not completely eliminate the insulin-stimulating activity, indicating that there are other substances with insulin-stimulating activity in the intestine (Drucker, 1998). In the 1980s, after identifying the cDNA sequence and mRNA of proglucagon, two new glucagon-like peptides, glucagon-like peptide-1 (GLP-1) and glucagon-like peptide-2 (GLP-2) (Bell et al., 1983; Lopez et al., 1983; Heinrich et al., 1984), were found in the additional sequence of proglucagon.

**FIGURE 1:**
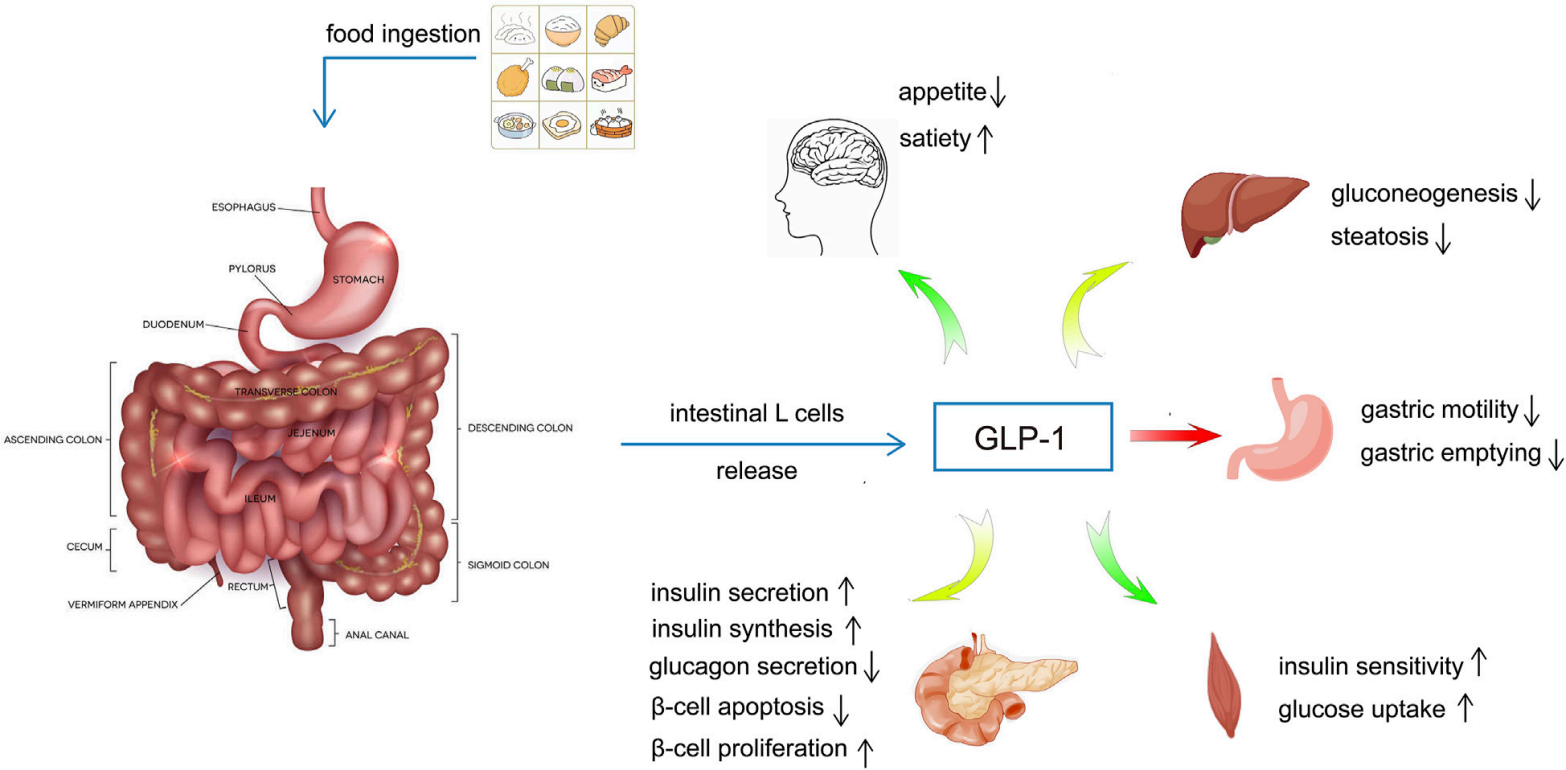
The regulatory effects of GLP-1 on blood glucose.

GLP-1 is a peptide hormone consisting of 30 amino acids derived from glucagonogen, which is processed by the differentiation of the glucagonogen gene expressed by intestinal epithelial endocrine L-cells, and has two biologically active forms, GLP-1 (7–36 amide) and GLP-1 (7-37), and the intestine is the major source of GLP-1 in plasma (Drucker, 1998; Holst, 2007). Carbohydrate, fat and protein intakes, as well as bile acids, can stimulate the secretion of GLP-1 by intestinal L-cells (Gribble and Reimann, 2021). GLP-1 has a variety of physiological functions, which can regulate blood glucose level by promoting insulin secretion and synthesis, stimulating β-cell proliferation and inducing islet regeneration, inhibiting β-cell apoptosis and glucagon release, delaying gastric emptying and controlling appetite (Müller et al., 2019; Gribble and Reimann, 2021; Drucker and Holst, 2023) (Figure 1). Since the discovery that GLP-1 can improve insulin secretion and glycemic control in patients with type 2 diabetes mellitus (T2DM) in the 1990s (Nauck et al., 1993; Ahrén and Gutniak, 1997; Vella et al., 2000), GLP-1 receptor agonists (GLP1-RAs) have been widely used in clinic.

T2DM is a highly prevalent chronic disease in the world, and its incidence has increased continuously in the past few decades, which has become a major public health problem (Glovaci et al., 2019; Lovic et al., 2020; Taylor et al., 2021). The high incidence and prevalence are attributed to the ageing population and poor lifestyles (Minari et al., 2023). GLP-1RAs, as an important class of drugs for the treatment of T2DM, shows significant advantages in blood glucose control, weight loss, cardiovascular protection and kidney benefit (Ussher and Drucker, 2023; Clark, 2024; Hu et al., 2024). Compared with the previously published articles, thecontents and purposes of this review are different: (1) Focusing on the therapeutic roles of GLP-1RAs in T2DM, the multiple pathways by which GLP-1 regulates blood glucose are described in a more comprehensive and in-depth manner at the mechanism level; (2) The action characteristics of various types of non-insulin hypoglycemic drugs are compared, as well as the advantages, shortcomings, points for attention and challenges of GLP-1RAs in treatment. It is expected that GLP-1RAs can be used more safely, effectively and reasonably for T2DM patients.

## 2 Influencing factors of GLP-1 secretion

GLP-1 is secreted by intestinal L cells and plays a key role in the regulation of blood glucose in a glucose-dependent manner (Zhang et al., 2023). The influencing factors of GLP-1 secretion mainly involve the following aspects: (1) Activation of related receptors. The expression of intestinal endocrine Lcells is regulated by several key receptors activated by short chain fatty acids (SCFAs), specific endogenous cannabinoids and bile acids, and the activation of these receptors increases the secretion of key intestinal peptides, such as GLP-1, GLP-2 and polypeptide YY (PYY) (de Vos et al., 2022). SCFAs receptors belong to the family of G protein-coupled receptors (GPCRs), including GPR41 and GPR43, which are important regulatory receptors for glucose metabolism and lipid metabolism, and can be activated by SCFAs to perform important physiological functions. SCFAs were able to increase the number of L cells in cultured intestinal cells *in vitro*, leading to an increase in GLP-1 release (Petersen et al., 2014). (2) Structural composition of the microbiota. The imbalance of gut microbiota will reduce the production of SCFAs, weaken the ability to activate SCFAs receptors, and affect GLP-1 secretion, resulting in abnormal glucose metabolism and lipid metabolism (Ma et al., 2019). (3) Dietary factors. In rats fed with high-fat diet, the density of intestinal epithelial cells producing GLP-1, secretory hormone and cholecystokinin reduced, and the expression of intestinal endocrine cells transcription factors encoded by Neurog3 and Neurod1 decreased accordingly (Sakar et al., 2014). In the primary culture cells of high-fat diet mice, transcriptome analysis showed that the gene expression of L cells with many characteristics was impaired (Richards et al., 2016). While a high-fiber diet was able to increase the density of L cells and proglucagon mRNA levels in the colon of obese and leptin-deficient mice (Everard et al., 2011). (4) Roux-en-Y gastric bypass surgery. It leads to a significant increase in postprandial GLP-1 and PYY concentrations in humans and animal models (Jørgensen et al., 2012; Peterli et al., 2012), which may be due to an increase in the number of L cells or in their secretion, or may reflect the flow of nutrients to more densely populated areas of L cells in the distal small intestine.

Sodium-glucose cotransport protein 1 (SGLT1) is a proximal glucose transporter responsible for absorbing glucose across the intestinal brush border, and it realizes glucose concentration gradient transport by coupling the uptake of each glucose molecule to the inward flux of two Na^+^ ions. This SGLT1-dependent mechanism is the basis for the early elevation of plasma GLP-1 and GIP levels after carbohydrate intake (Zhu et al., 2022). In mice lacking SGLT1, peak glucose-triggered GLP-1 and GIP levels were impaired (Gorboulev et al., 2012), and L cells showed no calcium response to glucose intake (Parker et al., 2012a). GPR119 is a Gαs-coupled receptor. Its activation leads to an increase in the concentration of cyclic adenosine monophosphate (cAMP) in target cells. cAMP is an efficient gastrointestinal hormone secretion stimulator (Gribble and Reimann, 2016; Zhang et al., 2024). Monoacylglycerol binds to GPR119 in L cells to promote the release of GLP-1, GIP and insulin (Chu et al., 2008). The G protein-coupled bile acid receptor (GPBA-R) is a membrane receptor coupled to Gαs signal transduction pathway. Bile acid stimulates GLP-1 secretion from GLUTag cells in a GPBA-R dependent manner. The elevated cAMP and Ca^2+^ were observed following GPBA-R activation in single GLUTag cell, and bile acid and GPBA-RA increased GLP-1 secretion in intestinal cultures (Parker et al., 2012b). Guanosine and uroguanosine, which target guanosine cyclase (GC-C) receptors in the intestine and kidney, lead to elevated intracellular cGMP concentrations, and high GC-C expression in L cells is associated with regulation of GLP-1 release (Friedlander et al., 2011). These substances may be closely related to the targets of action of GLP-1RAs for the treatment of T2DM.

## 3 Degradation of GLP-1 and production of GLP-1RAs

GLP-1 (7-36)NH_2_ acts as the active form, and the N-terminal end of this peptide is readily recognized by dipeptidyl peptidase-4 (DPP-4), an exopeptidase released from vascular endothelium and other tissues that can be quickly cut between the 2nd and 3rd amino acids to produce GLP-1 (9-36)NH_2_. The cycle half-life of GLP-1 (7-36)NH_2_ is 2–3 min, and the truncated metabolites are usually considered inactive. Approximately 90% of GLP-1 is metabolized by DPP-4 before reaching the central venous circulation (Holst and Deacon, 2005), and the peak circulating concentration of active GLP-1 (7-36)NH_2_ after a meal in healthy subjects is usually less than 10 pmol/L due to the rapid degradation of DPP-4 (Bak et al., 2014).

In order to solve the problem of rapid inactivation of GLP-1 by DPP-4 degradation, GLP-1RAs and DPP-4 inhibitors have been developed to treat T2DM (Kim and Jang, 2015; Böhm et al., 2017). GLP-1RAs have experienced a research and development process from short-acting to long-acting to ultra-long-acting, and from injection to oral administration. In 1992, exendin-4, which can stimulate insulin secretion, was isolated from *Heloderma suspectum* venom with 53% homology to mammalian GLP-1, and can resist DPP-4 degradation and has low renal clearance rate (Muttenthaler et al., 2021). The first GLP-1RAs Exenatide synthesized by exendin-4 was approved by the FDA in 2005 (Mariam and Niazi, 2024). Liraglutide is the second GLP-1RA to be approved after exenatide. Liraglutide is a human GLP-1 derivative, which is characterized by the retention of unaltered Ala at position 8, and structural modifications of amino acids at position 26 and 34 (Gong et al., 2024). Beinaglutide is currently the only GLP-1RA with 100% homology with human amino acid sequence (Du et al., 2020). Semaglutide’s introduction of a fatty acid side chain at position 26 Lys allows reversible binding to plasma albumin and reduces renal clearance, and substitution of amino acids at positions 8 and 34 with Aib and Arg avoids its degradation by DPP-4 and prevents fatty acid binding at the wrong site, resulting in a once-weekly dosing (Lau et al., 2015; Gong et al., 2024). The oral form prepared by Semaglutide and absorption enhancer SNAC (sodium N-[8-(2-hydroxybenzoyl) amino] caprylate), became the first oral GLP-1RA approved by the FDA for the treatment of T2DM (Kalra and Sahay, 2020; Aroda et al., 2022). GLP-1RAs can simulate the structure and function of GLP-1, thus play a role similar to GLP-1, and has a longer half-life, can persist and play a role in the body. The classification and usage of GLP-1RAs commonly used in clinic are shown in Table 1.

**TABLE 1:** Classification and usage of GLP-1RAs.

Drug name	Classification (Ji, 2022; Zeng et al., 2024)	Homology with human GLP-1 (%) (CSE and CDS, 2020; Zeng et al., 2024)	Half-life (Si et al., 2023; Zeng et al., 2024)	Initial dose (Zeng et al., 2024)	Maintenance dose (CSE and CDS, 2020; Si et al., 2023; Zeng et al., 2024)	Route of administration
Exenatide injection	Short-acting	53	2.4 h	5.0 μg, bid	5.0 μg/10.0 μg, bid	Hypodermic injection
Beinaglutide injection	Short-acting	100	11.0 min	0.1 mg, tid	0.1 mg/0.2 mg, tid	Hypodermic injection
Lixisenatide injection	Short-acting	50	3.0 h	10.0 μg, qd	20.0 μg, qd	Hypodermic injection
Liraglutide injection	Long-acting	97	13 h	0.6 mg, qd	1.2 mg/1.8 mg, qd	Hypodermic injection
Semaglutide injection	Ultra-long-acting	94	7.0 days	0.25 mg, qw	0.5 mg/1.0 mg, qw	Hypodermic injection
Dulaglutide injection	Ultra-long-acting	90	4.7 days	0.75 mg, qw	0.75 mg/1.5 mg, qw	Hypodermic injection
Exenatide microspheres for injection	Ultra-long-acting	53	2.4 h	2.0 mg, qw	2.0 mg, qw	Hypodermic injection
Polyethylene glycol loxenatide injection	Ultra-long-acting	50	144 ∼ 155 h	0.1 mg, qw	0.1 mg/0.2 mg, qw	Hypodermic injection
Semaglutide	--	94	7.0 days	3.0 mg, qd	7.0 mg/14.0 mg, qd	Oral administration

Note: qd: once a day; bid: twice a day; tid: three times a day; qw: once a week; CSE, Chinese Society of Endocrinology; CDS, Chinese Diabetes Society; -- indicates that the relevant information has not been found.

## 4 The mechanisms and pathways of GLP-1 regulating blood glucose

### 4.1 Promotion of insulin synthesis

Transcription factors regulate gene expression by binding to specific enhancer sequences. During the development and maturation of pancreatic β cells, key transcription factors include Pancreatic/duodenal homeobox 1 (Pdx1), neurogenin 3 (NEUROG3), and V-maf musculoaponeurotic fibrosarcoma oncogene homolog A (MafA) (Zhu et al., 2017). Pdx1, in particular, plays a critical role in the development of the pancreas and the function of β cells by binding to regulatory elements and promoting the transcription of the insulin gene (Yang et al., 2012). However, prolonged exposure of β cells to high-glucose environments can lead to decreased expression levels of Pdx1 and MafA, potentially inhibiting insulin biosynthesis and secretion (Kaneto and Matsuoka, 2015). Consequently, patients with T2DM often exhibit reduced Pdx1 expression levels (Ofori et al., 2022). Cordycepin has been found to promote insulin synthesis by upregulating the mRNA levels of insulin, Pdx1, and glucose transporter 1 (GLUT1), as well as the protein expression of Pdx1, GLUT1, Akt, and phosphorylated Akt (Sun et al., 2022).

GLP-1-induced insulin synthesis is initiated by Pdx1. On one hand, GLP-1 activates PKA, increasing the expression of Pdx1 and causing Pdx1 to translocate into the nucleus. Within the nucleus, Pdx1 binds to the insulin promoter, initiating insulin expression and synthesis (Wang et al., 2001; Müller et al., 2019). On the other hand, B-cell translocation gene 2 (BTG2) can upregulate Pdx1-induced insulin gene expression. Studies have shown that GLP-1 treatment significantly increases the expression levels of BTG2, Pdx1, and the insulin gene in pancreatic β cells, indicating that GLP-1 positively regulates insulin gene expression through BTG2, thereby promoting insulin synthesis (Hwang et al., 2013).

### 4.2 Promotion of insulin secretion

GLP-1 stimulates insulin secretion in a glucose-dependent manner through specific receptors expressed on pancreatic βcells.GLP-1R is a family of secretins (class B) that couples GLP-1 to downstream βcell responses, and conformational changes in the receptor upon ligand binding facilitate the interaction between its intracellular region and signaling proteins (Jones et al., 2018). GLP-1R is highly expressed in pancreatic βcells, providing a clear pathway to directly promote insulin secretion. After the knockout of the GLP-1R gene, in transgenic mice expressing human GLP-1R in pancreatic islets and pancreatic ductal cells restored cAMP and serine threonine kinase (Akt) phosphorylation levels in isolated islets, increased GLP-1-stimulated insulin secretion and GLP-1R-dependent β-cell proliferation, and promoted transgenic mice for glucose regulation in response to feeding (Lamont et al., 2012). β-cell-selective knockout of the GLP-1R in mice with impaired glucose tolerance after intraperitoneal injection of glucose and exogenous supplementation of GLP-1 did not induce insulin secretion, but glucose tolerance returned to normal after oral glucose, suggesting that the extra-islet GLP-1R plays a regulatory role in oral glucose tolerance (Smith et al., 2014). GLP-1 is able to stimulate cAMP formation in RIN 1046-38 cells, which in turn causes an increase in insulin mRNA levels and insulin release, and insulin accumulation in the culture fluid was observed by adding GLP-1 (7-37) to RIN 1046-38 cells for 1 h incubation (Drucker et al., 1987). In addition, GLP-1 (7-37) concentrations as low as 50 p.m. stimulated insulin release in perfused rat pancreas (Mojsov et al., 1987).

**FIGURE 2:**
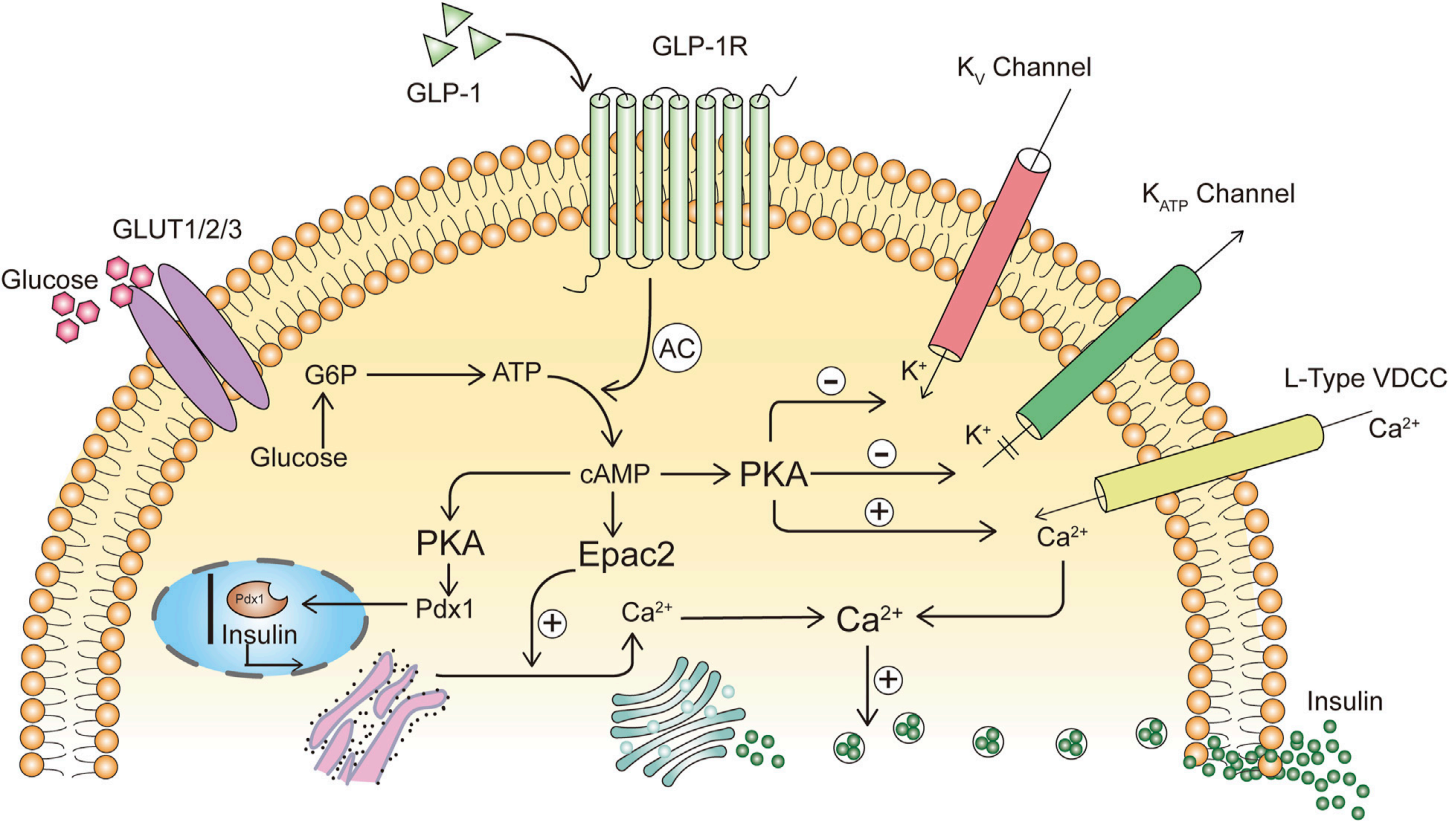
GLP-1 promotes insulin secretion.

The activation of the classical cAMP pathway is achieved by releasing irritating G protein subunit Gαs through the exchange of guanine nucleotide and the activation/dissociation of heterogeneous trimer G protein, and in pancreatic βcells, GLP-1 binds to the specific guanine nucleotide-binding protein-coupled receptor GLP-1R, leading to adenylyl cyclase activation and elevated cAMP levels (Mayo et al., 2003; Dalvi et al., 2012). cAMP is an important second messenger driving the acute insulin effect of GLP-1. Overexpression of phosphodiesterase 3B promotes cAMP hydrolysis and reduces GLP-1-induced insulin secretion, an effect evident in both isolated islets and cultured insulinoma cells (Härndahl et al., 2002). cAMP elevation induced by GLP-1 can lead to its downstream protein kinase A (PKA) activation and enhanced signaling via cAMP-activated guanine nucleotide exchange factor (Epac) (Holz, 2004; Doyle and Egan, 2007). cAMP activates PKA, which will phosphorylate the β2 subunit of the L-type VDC channel and the Kir6.2 and SUR1 subunits of the K_ATP_ channel, increasing K_ATP_ channel sensitivity to ATP and leading to K_ATP_ channel closure, cell membrane depolarization and VDC channel opening, followed by Ca^2+^ influx through VDC channels into βcells to promote the exocytosis of insulin granules and the acute secretion of insulin and into circulation (Bünemann et al., 1999; Müller et al., 2019) (Figure 2). GLP-1-activated PKA inhibits voltage-gated potassium channels, prevents membrane repolarization, and prolongs VDC channel opening to promote Ca^2+^ inward currents, contributing to GLP-1 stimulate insulin secretion, and the antagonistic effect of GLP-1R activation on Kv currents in βcells requires transactivation of epidermal growth factor receptors, accompanied by signal conduction of cAMP/PKA and phosphatidylinositol-3-kinase (PI3K)/PKCζ signaling (MacDonald et al., 2003). Both Epac1 and Epac2 are expressed in the pancreas (Leech et al., 2000), and under high glucose conditions, Ca^2+^ flows into βcells through VDC channels, and Epac2 opens RYR calcium channels in the endoplasmic reticulum, further increasing intracellular Ca^2+^ concentrations and enhancing exocytosis of insulin-secreting vesicles (Holz, 2004; Doyle and Egan, 2007). In isolated perfused rat pancreas, GLP-1 did not stimulate insulin release at glucose concentrations below 2.8 mM and could only promote insulin release when glucose concentrations were above 6.6 mM (Weir et al., 1989). As a result, compared with other hypoglycemic drugs, GLP-1RAs has a lower risk of hypoglycemia. In human islets, GLP-1 promotes increased connectivity between individual βcells to improve calcium kinetics and insulin secretion throughout the islets, and promotes insulin secretion from islet cells when βcells detect both elevated blood glucose levels and hormonal signals from the intestine.

### 4.3 Stimulation of β cell proliferation and induction of islet neogenesis

The proliferation potential of pancreatic β cells is very limited. Under normal conditions, new β cells generated through mitosis in adult rats constitute about 3% per day, a rate that rapidly declines with age (Swenne, 1983). However, *in vitro*, certain nutrients and growth factors can enhance their replicative activity. The size of β cell clusters is a major determinant of the total insulin secretion by the pancreas. Expanding β cell clusters through recruitment of β cell proliferation could help maintain normal blood glucose levels, potentially compensating for β cell loss or dysfunction in diabetes (Sjöholm, 1996).

**FIGURE 3:**
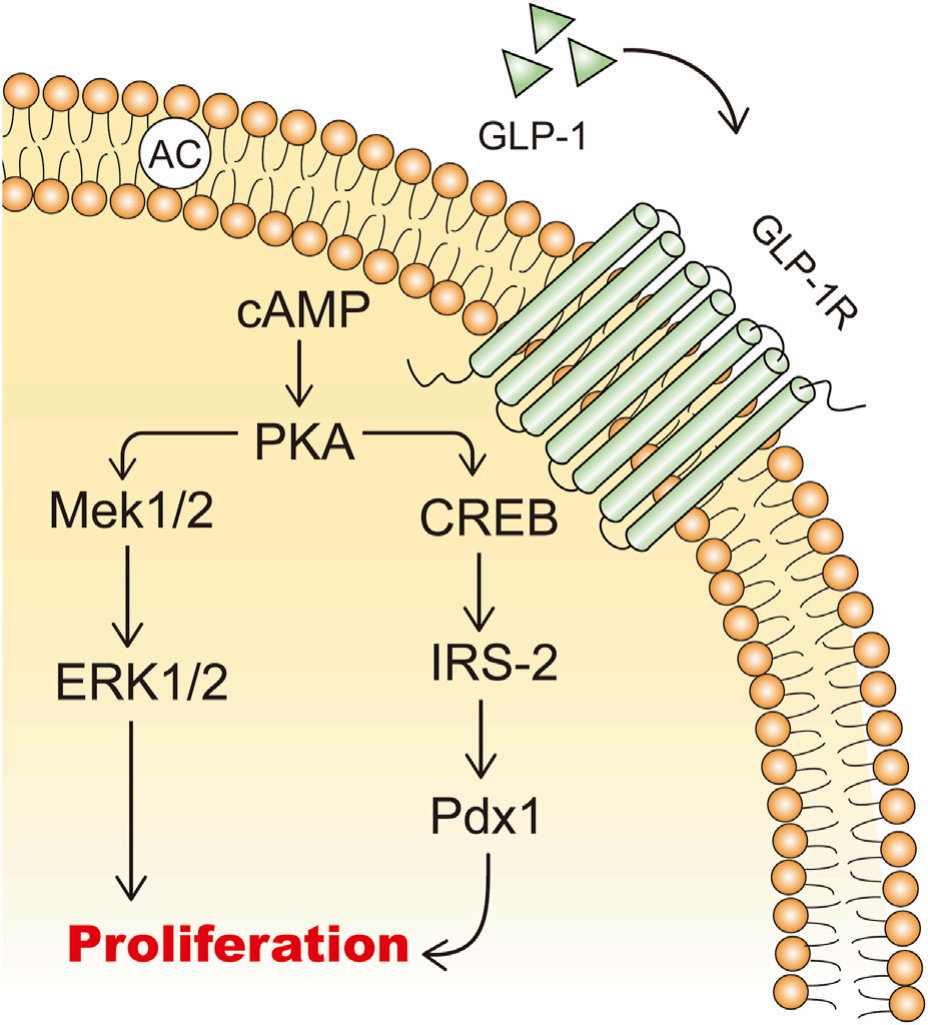
The mechanisms of GLP-1 stimulating β cell proliferation. IRS-2: insulin receptor substrate 2; Mek: mitogen-activated extracellular signal-regulated kinase; ERK: extracellular signal-regulated kinase.

GLP-1 and GLP-1RAs promote the expansion of β-cell mass by stimulating β-cell proliferation, inducing islet neogenesis, and inhibiting β-cell apoptosis (Wajchenberg, 2007). In isolated rat islets, GLP-1 acts as a growth factor in a dose-dependent manner to increase the incorporation of tritiated thymidine and promotes cell proliferation through the upregulation of PI3K-mediated pathways (Buteau et al., 1999). cAMP is a potent mitogenic factor for β cells, and GLP-1 increases intracellular cAMP levels, thereby promoting β-cell proliferation (Sjöholm, 1996; Susini et al., 1998) (Figure 3). GLP-1 promotes islet neogenesis, lowers blood glucose levels, upregulates Pdx1 expression, and increases β-cell mass in db/db mice (Stoffers et al., 2000). Furthermore, endogenous GLP-1 receptor signaling is crucial for the adaptive proliferative response of islets to metabolic stress and pancreatic injury (Drucker, 2003). In a partially pancreatectomized model of type 2 diabetic rats, continuous treatment with GLP-1RAs for 10 days mitigated the progression of diabetes (Xu et al., 1999). Therefore, GLP-1RAs hold promise as a novel therapeutic approach for diabetic patients with reduced β-cell mass, stimulating β-cell proliferation and differentiation (Iqbal et al., 2021).

### 4.4 Inhibition of β-cell apoptosis

Apoptosis plays an important role in the pathophysiology of diabetes. The reduction in β-cell mass in patients with T2DM can be attributed to β-cell apoptosis triggered by multiple factors, including islet amyloid deposition, lipotoxicity, glucotoxicity, and inflammation (Costes et al., 2021). Stress pathways induced by these factors, including endoplasmic reticulum stress, oxidative stress, mitochondrial dysfunction, and autophagy, are all involved in the induction of β-cell apoptosis, and these pathways may occur simultaneously at the same level or may interact to further exacerbate β-cell death in the form of superimposed harmful stress. In addition, some non-coding RNAs, such as Bcl-2, Ago, MafA, Pdx1, MTPN, and caspase-3, etc., also play an important role in β-cell apoptosis in T2DM patients by regulating the expression of key β-cell factors (You et al., 2022). β-cell apoptosis is mediated by a series of cascade mechanisms of the cysteine protease family, and current studies on hyperglycemia-induced β-cell apoptosis have focused on the balance between the pro-apoptotic Bcl-2 proteins (Bad, Bid, Bik, and Bax) and the anti-apoptotic Bcl family (Bcl-2 and Bcl-xl) (Tomita, 2016), and apoptosis occurs only when the concentration of the pro-apoptotic protein Bcl-2 on the mitochondrial membrane of the intrinsic pathway exceeds the concentration of the anti-apoptotic protein. In patients with T2DM, where islet neogenesis and β-cell replication are normal, increased apoptosis is the main cause of the decrease in their β-cell numbers, so preventing β-cell apoptosis may become an effective means of treating T2DM (Butler et al., 2003).

**FIGURE 4:**
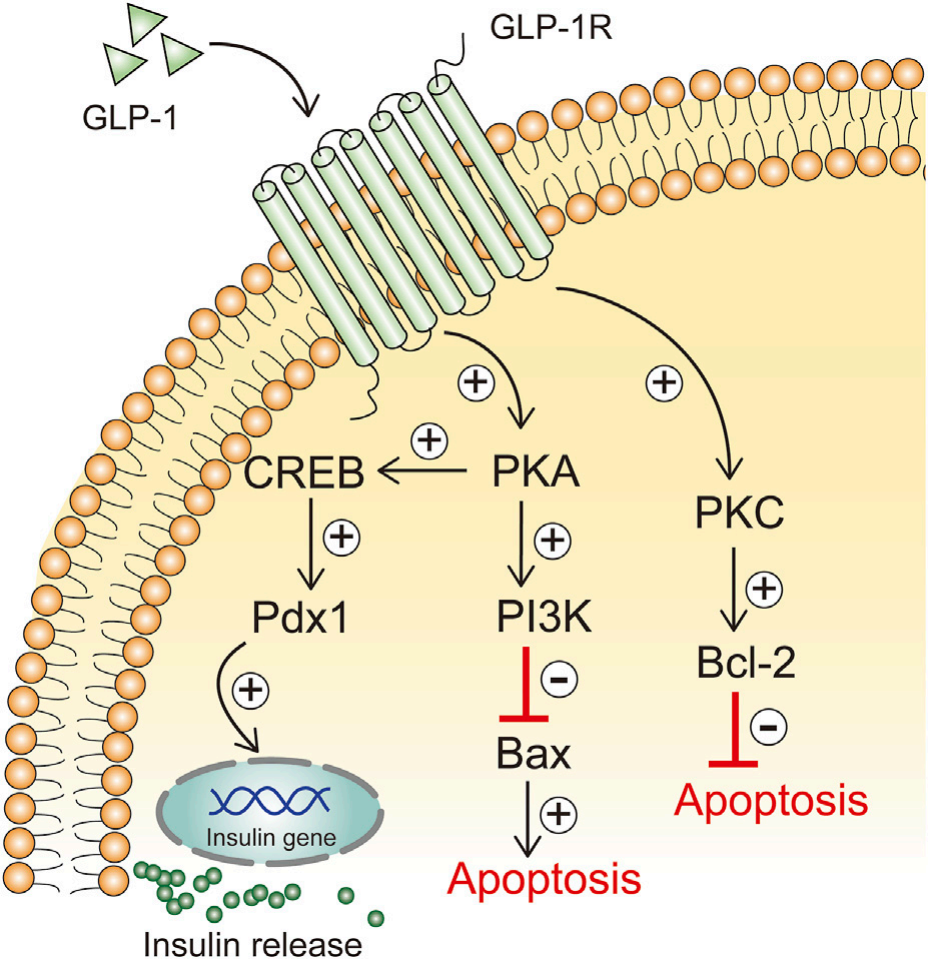
GLP-1 inhibits apoptosis of β cells.

GLP-1 has a beneficial effect on β-cell function by promoting β-cell proliferation and neogenesis and reducing apoptosis (Zheng et al., 2022). GLP-1 has anti-apoptotic effects *in vivo*, and its promotion of cells expressing the GLP-1 receptor directly resists β-cell apoptosis (Drucker, 2003). In addition, GLP-1 increases the activity of protein kinase C (PKC) in islet β cells in a dose-dependent manner, promotes pancreatic β-cell survival, and inhibits mitochondria-dependent apoptosis by regulating Bcl-2/Bax expression levels (Zhang et al., 2015). When GLP-1 binds to GLP-1R on the surface of β-cells, cAMP-dependent PKA is activated and subsequently activates the downstream signal PI3K, which inhibits apoptosis in islet β-cells by blocking the expression of the pro-apoptotic protein Bax (Li et al., 2021) (Figure 4). IKKε phosphorylates targets in the NF-κB signaling pathway, thereby activating the NF-κB pathway. NF-κB is an important nuclear transcription factor, which can promote the regulation of inflammation and apoptosis. The expression levels of IL-6, IKK-ε and NF-κB were elevated in pancreatic cells from T2DM rats, accompanied by increased Bax expression and decreased Bcl-2 expression, as well as decreased pancreatic β-cell function; The GLP-1 receptor agonist liraglutide was able to reduce Bax expression in pancreatic cells from T2DM rats, and Bcl-2 expression was increased, islet tissue structure was improved, insulin-positive expression was increased, and β-cell apoptosis was reduced (Liu et al., 2021). Liraglutide attenuates the inflammatory response by inhibiting IKK-ε/NF-κB, thereby reducing islet β-cell apoptosis.

### 4.5 Inhibition of glucagon release

**FIGURE 5:**
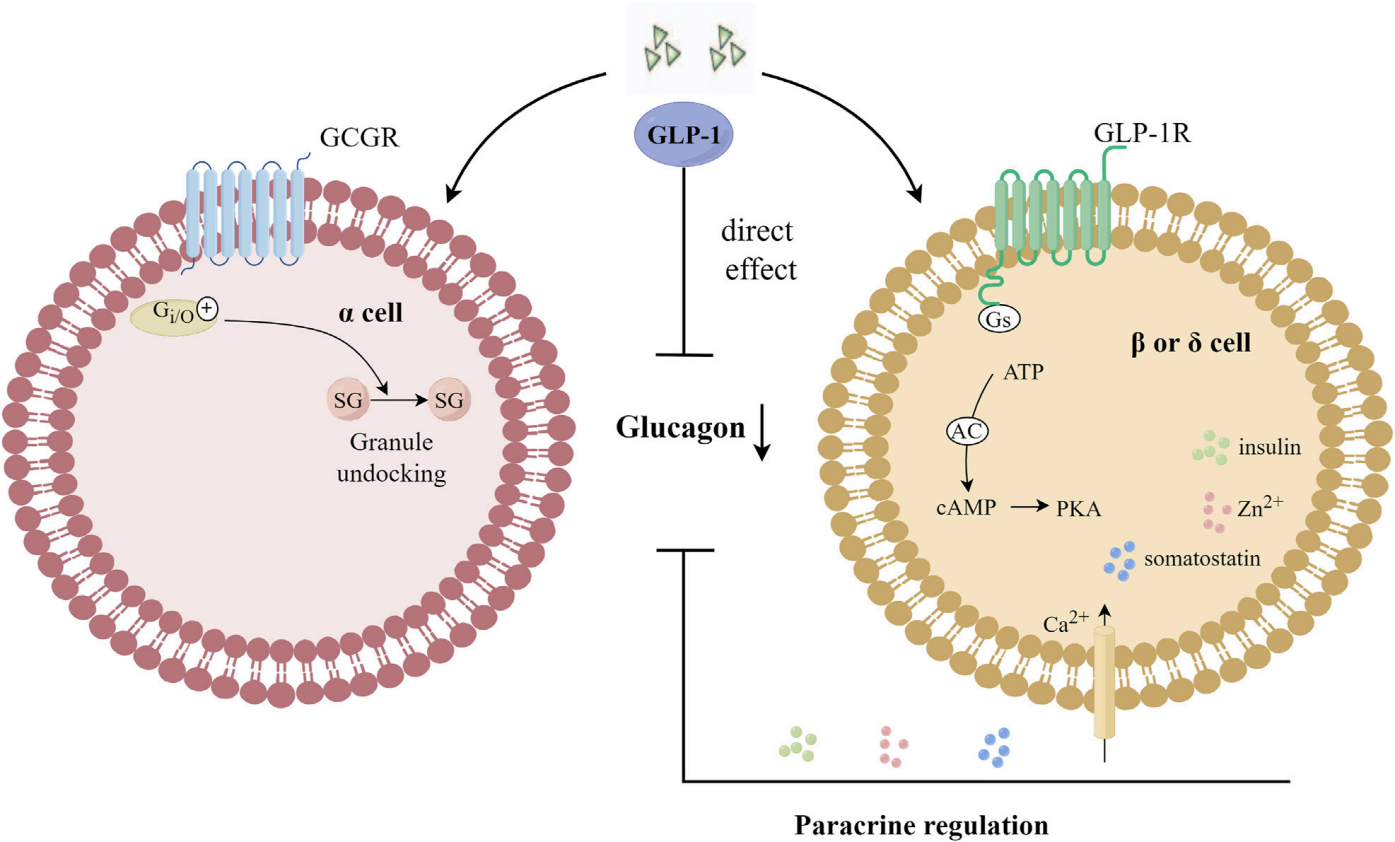
Mechanisms of GLP-1 inhibition of glucagon secretion. GLP-1 can activate GCGR in α cells, and GCGR is able to reduce the number of docking granules by coupling and activating Gi/o, thus inhibiting the secretion of glucagon. G_i/o_: an inhibitory GTP-binding protein; SG: secretory granules; GCGR: glucagon receptor.

Considered an anti-insulin hormone, 2022 marks the 100th anniversary of the discovery of glucagon, which is produced in islet α cells through the processing of prohormone-converting enzyme 2 (Pcsk2) and acts through glucagon receptor (GCGR) (Hayashi, 2011). Under normal physiological conditions, glucagon secreted by pancreatic islet α cells plays an important role in maintaining blood glucose homeostasis by regulating hepatic glucose production. Fasting and postprandial hyperglucagonemia in T2DM patients stimulates glucose production in the liver, leading to hyperglycemia. Inhibition of glucagon secretion or antagonism of glucagon receptors has become a potentially effective treatment strategy for T2DM patients (Lund et al., 2014). Both glucagon and GLP-1 receptors belong to the GPCR superfamily. Glucagon acts in the liver via cAMP (Abbas et al., 2020), and glucagon is secreted by α cells as an immediate response to glucose reduction. Compared with the saline group, the physiological dose of GLP-1 injected during the hyperglycemic clamp can significantly reduce the glucagon level in post-transplantation diabetic patients, suggesting that GLP-1 has a clear glucagon inhibitory effect and can restore the altered insulin and glucagon secretion in post-transplantation diabetic patients in a glucose-dependent manner (Halden et al., 2016). Giving exogenous GLP-1 analogues or increasing endogenous GLP-1 levels can enhance the signaling of GLP-1R, and the activation of GLP-1R can effectively inhibit glucagon secretion in human body. The inhibitory effect of GLP-1 on glucagon secretion *in vivo* was observed only when the blood glucose level was equal to or higher than the fasting level, at higher blood glucose levels, the inhibition of glucagon gradually increased (Holst, 2007), and when the blood glucose concentration was less than 3.7 m mol/L, GLP-1 lost its effect on inhibiting glucagon secretion (Nauck et al., 2002). GLP-1R expression on α cells is low. In pancreas, the main expression sites of GLP-1R are located in pancreatic β cells and δ cells (Richards et al., 2014), and the expression of GLP-1R in α cells was about 0.2% of that in β cells (De Marinis et al., 2010), or even absent (Tornehave et al., 2008). Therefore, indirect regulation, via β- or δ-cell products has been thought to be the primary mechanism by which GLP-1 inhibits glucagon secretion (Gromada et al., 2007; Christensen et al., 2011; Müller et al., 2019; Davis and Sandoval, 2020). However, GLP-1 can activate GCGR in α cells, and GCGR is able to reduce the number of docking granules by coupling and activating inhibitory GTP binding protein (Gi/o), thus inhibiting the secretion of glucagon, which is independent of GLP-1R (Gandasi et al., 2024). The mechanisms of GLP-1 inhibiting glucagon secretion are shown in Figure 5.

### 4.6 Delayed gastric emptying

GLP-1RAs has been shown to delay gastric emptying (Jelsing et al., 2012), thereby extending the retention of food in the stomach and small intestine and reducing postprandial glucose levels (Flint et al., 2011). GLP-1 is an energy sensor that reduces intestinal motility, prolonging the retention of food in the gut, contributing to more efficient absorption of nutrients and better bioavailability (Winkler et al., 2019). GLP-1 infusion slows gastric emptying of solid and liquid components of food and alters the distribution of food in the stomach so that a greater proportion of food is retained in the distal stomach (Marathe et al., 2011). Even intravenous administration of GLP-1 at a physiological postprandial dose of 0.3 pmol/kg/min significantly slowed the rate of gastric emptying in healthy subjects, bringing them into a state of “gastroparesis” (Little et al., 2006). Exogenous GLP-1 improves gastric compliance in a dose-dependent manner (Schirra et al., 2002), reduces the frequency and amplitude of gastric antrum and duodenum contraction, inhibits the contractile force of gastric antrum and duodenum, increases pyloric tension, and stimulates pyloric tetanic and phase motion (Schirra et al., 2000). Endogenous GLP-1 affects postprandial blood glucose by slowing gastric emptying and glucose absorption in healthy subjects (Deane et al., 2010) by inhibiting antral and duodenal motility and stimulating pyloric motility (Schirra et al., 2006). Other studies (Delgado-Aros et al., 2002) have shown that GLP-1 can increase fasting and postprandial stomach capacity and delay gastric emptying by inhibiting the cholinergic function of vagus nerve, without inducing postprandial symptoms. GLP-1 also helps with ileal braking, a circuit that regulates the rate of gastric emptying so that the rate of nutrients entering the duodenum is balanced with that of nutrients in the upper small intestine. If the flow of nutrients from the stomach is too fast, duodenum and jejunum absorption is incomplete, and the increased nutrient load in the ileum triggers increased GLP-1 and PYY secretion, which in turn feeds back regulation and further slows gastric emptying, thus restoring balance to the system. To sum up, the potential mechanisms of delayed gastric emptying by GLP-1 are mainly related to: (1) decreasing the contractile force of the gastric antrum and duodenum and increasing the pyloric tension (Schirra et al., 2000; Schirra et al., 2006); (2) inhibiting the cholinergic function of the vagus nerve (Delgado-Aros et al., 2002); (3) ileal braking (Layer et al., 1995; Holst, 2022).

### 4.7 Appetite and satiety

**FIGURE 6:**
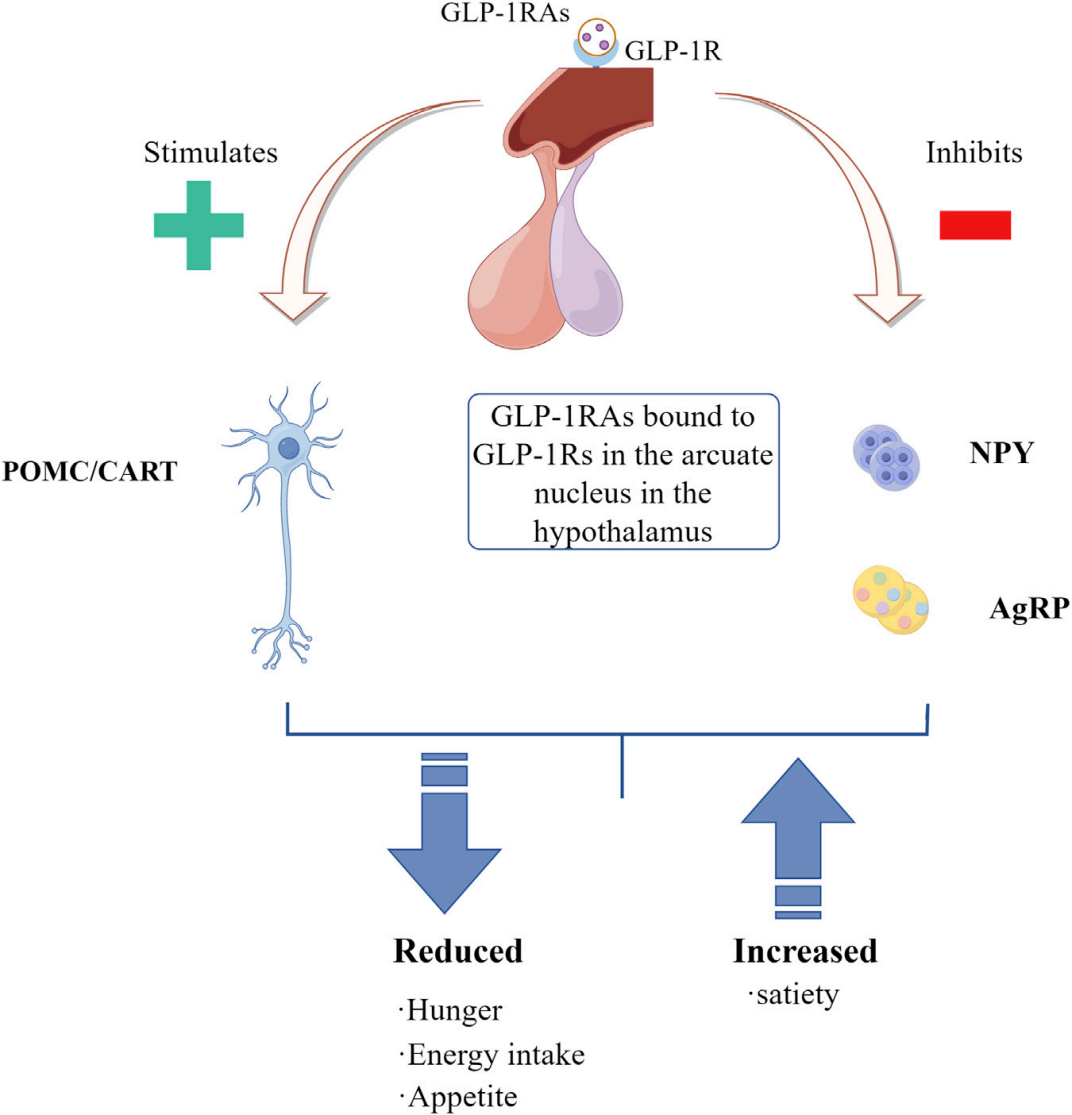
The roles of GLP-1RAs in the central nervous system.

Appetite and weight regulation are controlled by the central nervous system in a fairly complex manner, with the human brain playing a central role in integrating internal and external inputs and regulating energy homeostasis (Farr et al., 2016a). Farr et al. first demonstrated that GLP-1 receptors exist in the parietal cortex, hypothalamus and medulla of the human brain and are involved in regulating food intake (Farr et al., 2016b). GLP1-RAs is clinically used to treat T2DM and promote weight loss in obese individuals. Liraglutide can enter specific brain regions associated with appetite regulation, electrophysiological measurements of mouse brain sections showed that GLP-1 directly stimulated promelanocortin (POMC)/cocaine and amphetamine regulated transcript (CART) neurons and indirectly inhibited neurotransmission of neuropeptide Y (NPY) and agouti-related peptide (AgRP) in arcuate nucleus neurons through GABA-dependent signal transduction. GLP-1R on arcuate nucleus neurons expressing POMC/CART can mediate Liraglutide-induced weight loss (Secher et al., 2014). GLP-1RAs directly stimulate POMC/CART neurons and indirectly inhibit NPY and AgRP to increase measures of satiety and decrease hunger, resulting in reduced energy intake, thereby facilitating weight loss (Ard et al., 2021) (Figure 6). Consumption of high-calorie, high-sugar and high-fat foods is conducive to the development and maintenance of obesity (Berthoud and Zheng, 2012). Obese subjects were given liraglutide 3 mg once a day for 16 weeks. Compared with placebo group, liraglutide increased subjects’ satiety, reduced intake of sweet, salty and high-fat foods, and increased plasma PYY level after meals. As well as reduced fat storage throughout the body, trunk, and upper and lower body, these results suggest that liraglutide has a central role in altering appetite cravings (Kadouh et al., 2020). GLP-1RAs affects the central nervous system, including reducing appetite, improving diet control, increasing satiety, reducing calorie intake, etc. In general, GLP-1RAs provides a highly effective and well-tolerated treatment regimen to help obese patients lose weight while improving weight-related complications (Ard et al., 2021).

## 5 Application of GLP-1RAs in T2DM

GLP-1RAs can be divided into two categories: animal GLP-1 and its derivatives and human GLP-1 and its derivatives, which has experienced a development process from short-acting to long-acting to ultra-long-acting (Ji, 2022). Compared with placebo, GLP-1RAs treatment in T2DM patients significantly reduced the risk of death from cardiovascular disease and fatal/non-fatal stroke in cardiovascular outcome trials (Qin and Song, 2022). GLP-1RAs contributes to weight loss and lower glycosylated hemoglobin (HbA1c), and has a renal protective effect and is preferred in patients with higher cardiovascular risk T2DM and those considering weight loss (Ng et al., 2022). In addition, GLP-1RAs also have the effects of anti-inflammation, neuroprotection, lowering blood pressure, improving blood lipid profile and protecting liver (CSE and CDS, 2020; Zhao et al., 2021; Ajabnoor et al., 2023) and can benefit specific subgroups of T2DM patients: T2DM patients at risk of cardiovascular disease, kidney disease, non-alcoholic fatty liver disease, cognitive impairment, etc. Compared with other non-insulin hypoglycaemic agents, GLP-1RAs have the advantage of lowering HbA1c by a large margin and improving cardiovascular system and renal function without causing hypoglycaemia and weight gain (Table 2). In GLP-1RAs, Semaglutide is better than Dulaglutide in lowering HbA1c concentration (Chen et al., 2023). And Semaglutide effectively reduces low-density lipoprotein and total cholesterol levels, and performs best in weight loss (Xie et al., 2023; Yao et al., 2024).

**TABLE 2:** Characteristics of the action of non-insulin hypoglycaemic drugs.

Classification	Main mechanism	Lowering HbA1c (%) (CSE, 2018; Zeng et al., 2024)	Whether hypoglycemia occurs (Gao et al., 2014; CSE, 2018; Liu and Zhu, 2022)	Effect on body weight (Gao et al., 2014; CSE, 2018; Liu and Zhu, 2022)	Effects on cardiovascular and kidney (Gao et al., 2014; CSE, 2018; Liu and Zhu, 2022)	
					Cardiovascular system	Kidney disease
Metformin	Reduce the production of liver sugar, inhibit the intestinal absorption of glucose and improve the sensitivity of insulin	1.0 ∼ 1.5	no	Mild weight loss	Potential benefit	Neutral
α-glycosidase inhibitor	Delay the degradation of sugars in the intestinal tract	0.5 ∼ 1.0	no	Lose weight/neutral	Neutral	Neutral
Sulfonylureas	Promote insulin secretion	1.0 ∼ 1.5	yes	Gain weight	Neutral	Neutral
Glinides	Promote insulin secretion	0.5 ∼ 2.0	yes	Gain weight	Neutral	Neutral
Thiazolidinediones	Improve insulin resistance	0.7 ∼ 1.0	no	Gain weight	May cause/aggravate HF, forbidden in patients with NYHA cardiac function class Ⅱ or above	Neutral
DPP-4 inhibitor	Inhibition of GLP-1 degradation *in vivo*	0.4 ∼ 0.9	no	Neutral	May increase the risk of HF hospitalization	Neutral
SGLT-2 inhibitor	Inhibition of glucose reabsorption in urine	0.5 ∼ 1.5	no	Lose weight	Benefit	Benefit
GLP-1RAs	Activation of GLP-1 receptor	0.9 ∼ 1.8	no	Lose weight	Benefit	Benefit

Note: SGLT-2, sodium-dependent glucose transporters 2; HF, heart failure; NYHA, New York Heart Association.

However, as polypeptide drugs, GLP-1RAs are easily hydrolyzed by enzymes in the gastrointestinal environment. Coupled with their high molecular weight and high polarity, it is usually difficult to penetrate the gastrointestinal epithelial cell membrane, resulting in low oral bioavailability. Most of the GLP-1RAs on the market are injection forms, and the development of oral dosage forms is very important to shape the potential of GLP-1RAs market in the future. Common side effects of GLP-1RAs include gastrointestinal reactions such as nausea, vomiting and diarrhea (Chin et al., 2023; Aldhaleei et al., 2024), which may lead to a decline in the quality of life and even lead to drug withdrawal in some patients. Although the risk of hypoglycemia in GLP-1RAs is low, when used in combination with other hypoglycemic drugs or improper dose adjustment, patients may still have symptoms of hypoglycemia (Wolffenbuttel et al., 2023). In addition, long-term use of GLP-1RAs may also have potential safety risks, such as pancreatitis (Shetty et al., 2022) and thyroid diseases (Bezin et al., 2023), which require continuous monitoring and evaluation.

## 6 Discussion

T2DM is a chronic disease characterized by hyperglycemia, which eventually leads to microvascular damage and macrovascular events, as well as complications related to peripheral vascular diseases. Patients with obesity, hypertension, lipid disorders and various other risk factors have significantly increased rates of complications (Nauck et al., 2021b). In addition to a healthy lifestyle and prevention and treatment specifically aimed at diabetes-related complications, blood glucose control remains the primary approach to T2DM management.

GLP-1RAs may be the drugs of choice for the treatment of diabetes because of their cardioprotective, neuroprotective and renal protective activities other than lowering blood glucose, while not causing side effects of hypoglycemia and weight gain, with a proven efficacy and safety (Sharma et al., 2018; Nauck et al., 2021a). Other benefits of ultra-long-acting GLP-1RAs include less fluctuation in blood concentration, improved gastrointestinal tolerance, and a simpler and more convenient once-a-week dosing regimen that improves the compliance of patients and the persistence of treatment (Gentilella et al., 2019). Although a large number of short-term and medium-term clinical studies support the safety and efficacy of GLP-1RAs, there are insufficient data on the presence of delayed side effects with their long-term (decades) use (Guirguis et al., 2024). The response of different patients to GLP-1RAs may be different, and some patients may not be able to achieve the expected hypoglycemic effect. This may be related to genetic background, disease progression, islet function, obesity, lifestyle and other factors. It is a challenge to predict the individual response to GLP-1RAs through accurate medical treatment and optimize the treatment plan accordingly. T2DM patients are often accompanied by other comorbidities and need to take multiple drugs at the same time. The interactions between GLP-1RAs and other drugs may affect drug efficacy or increase the risk of adverse reactions, as well as the dose adjustment of the combination of hypoglycemic drugs, which put forward higher requirements for clinicians. GLP-1RAs usually belong to biological agents, and the production processes are complex, resulting in relatively expensive drug price. For some patients, especially those who do not have adequate medical insurance coverage, long-term use of such drugs may impose a heavy financial burden. Therefore, it is particularly important to reduce the costs of GLP-1RAs research and development.

Overall, GLP-1RAs is a relatively safe and effective new hypoglycemic drug, which helps to delay the occurrence and development of diabetes. At present, several kinds of intestinal hormone co-agonists are under development and have made progress through clinical trials. GLP-1-GIP co-agonist tirzepatide was approved by the US Food and Drug Administration in 2022 for the treatment of T2DM (Nogueiras et al., 2023). With the gradual deepening of the research and the continuous improvement of clinical status, GLP-1RAs brings new hope for the comprehensive treatment of T2DM patients.

## Abbreviations

GLP-1, glucagon-like peptide-1; GLP-1R, glucagon-like peptide-1 receptor; GLP-1RAs, glucagon-like peptide-1 receptor agonists; T2DM, type 2 diabetes mellitus; GIP, glucose-dependent insulin-stimulating peptide; GLP-2, glucagon-like peptide-2; GCGR, glucagon receptor; DPP-4, dipeptidyl peptidase-4; SCFAs, short chain fatty acids; GPCRs, G protein-coupled receptors; PYY, polypeptide YY; SGLT1, sodium-glucose cotransport protein 1; cAMP, cyclic adenosine monophosphate; GC-C, guanosine cyclase; Akt, serine threonine kinase; PKA, protein kinase A; Epac, guanine nucleotide exchange factor; PI3K, phosphatidylinositol-3-kinase; Pdx1, Pancreatic/duodenal homologous protein 1; NEUROG3, neurogenin 3; MafA, V-maf musculoaponeurotic fibrosarcoma oncogene homolog A; GLUT1, glucose transporter protein 1; BTG2, B-cell translocation gene 2; PKC, protein kinase C; POMC, promelanocortin; CART, cocaine and amphetamine regulated transcript; HbA1c, glycosylated hemoglobin.
